# Microbial Community Composition and Major Environmental Factors Influencing Changes in Different Vegetation Soils of Coastal Wetlands

**DOI:** 10.3390/microorganisms14020497

**Published:** 2026-02-19

**Authors:** Dongmei He, Weixiang Liu, Lei Wang, Wenwen Xu, Jiaojiao Zhang, Qihang Lu, Ting Luo

**Affiliations:** 1 Jiangsu Academy of Forestry, Nanjing 211153, China; ybfqxy@126.com (D.H.);; 2 Jiangsu Yancheng Coastal Wetland Ecosystem Positioning Observation and Research Station, Yancheng 224000, China; 3 School of Environmental Science and Engineering, Yancheng Institute of Technology, Yancheng 224051, China; 4 Jiangsu Province Ecology and Environment Protection Key Laboratory of Ecology and Pollution Control of Coastal Wetlands, Yancheng 224051, China

**Keywords:** soil microbial community, environmental factors, alpha diversity, coastal wetlands

## Abstract

The soil microbial community in coastal wetlands plays a crucial role in biogeochemical cycling. In this study, *Spartina alterniflora* soil (HB), found near the sea; *Spartina alterniflora* soil (ZY), found near land; and *Phragmites australis* soil (KA), found in coastal wetlands, were selected to study the microbial community structure and major environmental influencing factors. The results showed that environmental factors had a significant difference in the three soils. Compared with the ZY and KA sites, the soil at the HB site had the highest value of salinity (14.1 g/kg) and the lowest value of total organic carbon (TOC) (2.9 g/kg) in summer. At the KA site, the values of soil temperature, soil humidity (SH), TOC, and NH_4_^+^ were higher than those at HB and ZY sites, while the values of EC (159.8 μS/cm in summer) and salinity (4.4 g/kg) were the lowest. Furthermore, the microbial community structure had significant differences at the three sites. *Pseudomonas* and *Bacteroidota* dominated at the HB site, while *Chloroflexota* and *Gemmatimonades* were more abundant at the ZY and KA sites. Microbial alpha diversity analysis indicated that the microbial community diversity of *Phragmites australis* soil was the most uniform, and the microbial species richness in the soil of *Spartina alterniflora* near the sea was the highest. Salinity, TOC, and SH might be the key environmental factors that affect the structure and diversity of microbial communities in soils. High-salt environments may promote the enrichment of salt-tolerant microbial communities, while high TOC and suitable soil humidity may enhance the uniformity of microbial communities.

## 1. Introduction

Coastal wetland areas represent some of the most important natural ecosystems. The coastal mudflat wetland is an example of a coastal wetland ecosystem in China [1]. The coastal mudflat wetland comprises various habitats, such as intertidal zones and salt marshes, and is also a key stopover for migratory birds. The microbial community in coastal wetland soils is a key driver of material cycling and energy flow in wetland ecosystems [2]. However, in recent years, coastal wetlands have encountered various environmental changes, such as sea level rise, hydrological changes, pollutant runoff, and eutrophication [3]. Therefore, the composition of microbial communities in coastal wetland soils and major environmental influencing factors have important implications for microbial action in wetland ecosystems.

Coastal wetland vegetation is a key link connecting land and marine ecological processes. Vegetation regulates the spatiotemporal distribution of key environmental factors, such as soil temperature, salinity, total organic carbon (TOC), and nitrogen forms, by intercepting precipitation, reducing tidal erosion, root exudates, and physiological metabolism [4,5]. Microorganisms participate extensively in the carbon cycle, as well as the cycling of nitrogen, and other elements, in wetland environments, affecting the carbon storage and greenhouse gas emissions of wetland ecosystems [6]. The adaptability and dynamic changes in microorganisms can effectively reflect the sensitivity and resilience of wetland ecosystems to environmental changes [7]. Temperature is an important environmental factor that affects the metabolic activity of microorganisms, and seasonal changes in temperature can directly impact on bacterial community diversity and function [8]. Studies have shown that temperature and nitrite are the main environmental factors affecting the seasonal variation in bacterial communities in the Yellow River Delta [9]. Changes in soil humidity can also influence the structure of microbial communities, and sufficient water is beneficial for the survival of microorganisms. Zhou et al. [10] reported that the composition of microorganisms will shift towards being dominated by bacteria under conditions of moderate humidity. Salinity also has an impact on the structure of microbial communities. Microbial community diversity is usually high in low-salt wetland environments, and these microorganisms can adapt to relatively low ion concentration environments [11]. Zhang et al. found that increasing salinity leads to an increase in bacterial abundance by comparing saline soils in the intertidal and supratidal zones with different salinity gradients [12], indicating the crucial role of salinity on bacteria. Furthermore, TOC is also a key driving factor for changes in microbial community structure in soils contaminated with heavy metals [13]. Nitrogen is an essential element that microorganisms need to synthesize key biomolecules such as proteins and nucleic acids. It not only directly impacts the growth and reproduction of microorganisms but also regulates structural composition and nitrogen cycling. In extreme oligotrophic environments, the availability of nitrogen has crucial implications for microbial community activity. Zhang et al. found that the microbial diversity and nitrogen fixation gene abundance of monsoon-dominated glaciers were significantly higher than those of westerly wind-dominated glaciers [14]. High nitrate contents in westerly wind-dominated glaciers increased the abundance of denitrification genes, clearly demonstrating the regulatory effect of nitrogen on glacier microbial community structure and function. Therefore, environmental factors, including temperature, soil humidity, TOC, salinity, and the different forms of nitrogen, may significantly affect microbial composition in wetland soils.

The coastal wetlands in Yancheng, Jiangsu Province are located in the core area of the west coast of the Yellow Sea. The typical vegetation of *Spartina alterniflora* and *Phragmites australis* has a clear distribution, and its ecosystem dynamics are related to tidal activities of the Yellow Sea. The tides in tidal flat wetlands cause the soil to alternate between seawater inundation and exposure to air drying. During high tide, the Yellow Sea carries a large amount of dissolved salts and suspended organic matter into the wetlands, causing a rapid increase in soil salinity in coastal areas. Seawater infiltration gradually decreases in the area extending inland, resulting in high salinity near the sea and low salinity inland. Previous studies have mostly focused on single environments. For example, some studies have targeted offshore salt marsh ecosystems dominated by *Spartina alterniflora*, emphasizing the effects of high salinity, frequent tidal inundation, and strong marine influence on microbial communities [12,15]. These studies revealed the enrichment of salt-tolerant microbial groups, such as *Pseudomonadota*, in offshore environments. Several studies have focused on nearshore or inland wetland ecosystems dominated by *Phragmites australis*, specifically examining terrestrial climate regulation’s effect on microbial diversity and element cycling [16,17]. However, there is still a lack of studies on the changes in soil microbial communities amidst different vegetation types in coastal mudflat wetland areas (such as the coastal mudflat wetland area of Yancheng), and research comparing offshore and nearshore environments is especially needed. The knowledge of how microbial communities respond to gradual environmental gradients (e.g., salinity, TOC, humidity) from offshore areas to nearshore areas remains limited.

Therefore, the objectives of this study were (1) to investigate the changes in physicochemical properties, such as temperature, soil humidity, salinity, NO_2_^−^, and NO_3_^−^, in soils of wetlands with different vegetation types; (2) to study the differences in microbial community structure and composition in wetland soils using metagenomic sequencing; and (3) to explore the main environmental factors that affect the structure of soil microbial communities amidst different types of vegetation.

## 2. Materials and Methods

### 2.1. Description of the Sampling Sites

**Figure 1:**
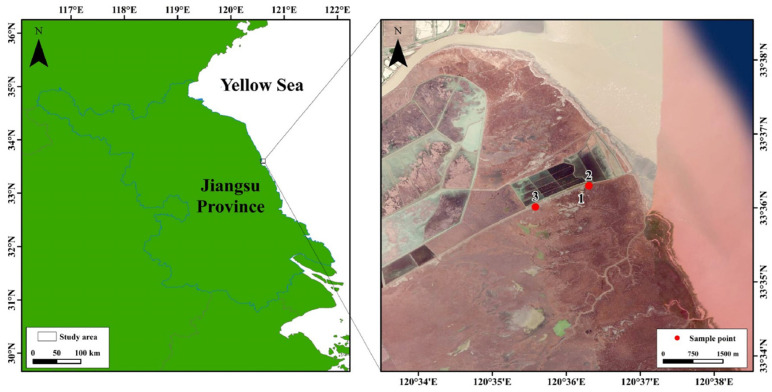
The location of sampling sites in coastal wetlands. HB: *Spartina alterniflora* soil, found near the sea. ZY: *Spartina alterniflora* soil, found near the land. KA: *Phragmites australis* soil.

The study site is located in the Sheyang tidal flat wetland in Yancheng City. Sheyang County (119°55′48″ E~120°34′47″ E, 33°31′12″ N~34°07′15″ N) is located in the central coastal area of Jiangsu Province, bordering the east side of the Yellow Sea. The inland beach area is three square kilometers, and the coastal beach area is 118 square kilometers. It is a typical muddy coastal wetland with multiple habitats, including an intertidal zone and a salty marsh area. The local microbial community is highly diverse and complex, and this is reflected in the ecological characteristics and sea–land interactions. Meanwhile, the region has a significant salinity gradient, periodic tidal hydrological dynamics, and vegetation zonation. Three sampling sites with different vegetation types were outlined, named HB, ZY, and KA, respectively (Figure 1). HB represents *Spartina alterniflora* soil, found near the sea; ZY represents *Spartina alterniflora* soil, found near the land; and KA represents *Phragmites australis* soil. The three sampling sites stretched from the sea to the inland.

### 2.2. Soil Sample Sampling

Sampling was carried out in July, August, September, November, and December 2024 and January 2025; the selected months covered the seasons of summer, autumn and winter. On the 1st, 15th, and 30th of each month, a monitoring electrode (SMEC300, Anasys, Santa Barbara, CA, USA) was used to measure soil temperature (ST), soil humidity (SH), and soil conductivity (EC) in situ. Soil surface samples (0–20 cm) were collected in January 2025. This depth of the soils contains part of the root systems of *Spartina alterniflora* and *Phragmites australis* [17], exhibiting the high microbial diversity and activity [5]. The soil samples were stored in cold conditions (4 °C), before being transported back to the laboratory. Some of the soil samples were air-dried and sieved (100 mesh) in the laboratory to analyze the physicochemical properties of the soil. The others were frozen and used for microbial metagenomic sequencing. All sample analyses were conducted in duplicate.

### 2.3. Analysis of Soil Physicochemical Properties

The concentrations of NO_3_^−^, NO_2_^−^, and NH_4_^+^ in the soil samples were determined via spectrophotometry [18]. Total organic carbon (TOC) content was analyzed by potassium dichromate oxidation method [19]. The soil solution was prepared according to a water–soil ratio of 1:2.5, and pH values were determined using a monitoring electrode [20]. Soil salinity was determined by gravimetric method [21].

### 2.4. Metagenomic Sequencing

Frozen soil samples were used for metagenomic analysis. Microbial DNA from frozen soil samples was extracted using the E.Z.N.A.^®^ Stool DNA Kit (Omega Bio-tek, Norcross, GA, USA). The detailed procedures were as follows: For each sample, 1 μg of genomic DNA was fragmented using a Covaris S220 focused ultrasonicator (Covaris, Woburn, MA, USA), and DNA fragments of approximately 450 bp were selected for sequencing library construction. All samples were sequenced on an Illumina Novaseq 6000 high-throughput sequencer (Illumina, San Diego, CA, USA), operating in paired-end 150 bp (PE150) sequencing mode to generate raw data. Raw sequencing data were quality-controlled using Trimmomatic (v.0.39) to remove adapter-contaminated sequences and low-quality reads. The remaining high-quality sequences (clean data) were used as valid data for subsequent analyses. The method used has been previously described [22].

### 2.5. Statistical Analysis

ArcGIS Pro software (v.3.4.3, 2024) was used to obtain a geographical map of the coastal wetland area. A nonmetric multidimensional scaling (NMDS) analysis was performed, based on Bray–Curtis distance, using R software (v.3.6.1; R Core Team, 2019). Relative abundance plots of nitrogen and carbon cycle functional genes were plotted using Origin (v.9.8.0, 2021). A correlation heat map between microbial alpha diversity index and environmental factors was established using the R software packages (vegan, ggplot2, v.4.1.1).

## 3. Results and Discussion

### 3.1. Changes in ST, SH, and EC in Different Vegetation Soils

**Figure 2:**
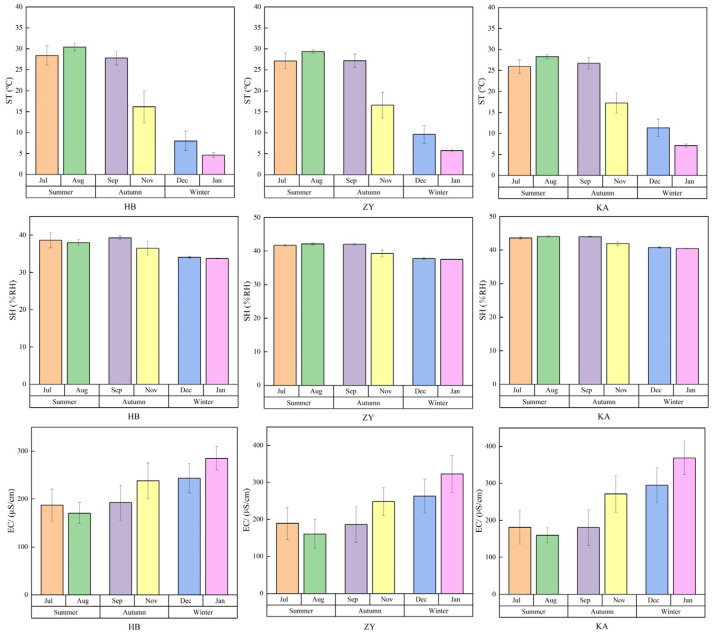
Seasonal variation in soil temperature (ST), soil humidity (SH), and soil electrical conductivity (EC) at the three sites in coastal wetlands.

The ST, SH, and EC values of the different soils in the tidal flat wetland area are shown in Figure 2. Among the three sites, the soil ST at the HB site was the highest in summer, and it reached the highest value of 30.4 ± 0.9 °C in August. In winter, the soil ST was the lowest, reaching its lowest value in January (4.6 ± 0.5 °C) at the HB site. At the KA site, the soil ST reached its highest value, 11.3 ± 2.1 °C, in December. The HB site is close to the sea and is surrounded by seawater year-round. The fact that this seawater-surrounded site is subjected to direct sunlight may explain the high temperature in summer. However, the temperature is regulated by the sea, and the water temperature cools more slowly than that on land in winter. Therefore, the soil temperature at the HB site is relatively low in winter. Bu et al. studying the Dongtan wetland of the Yangtze River estuary, found that *Spartina alterniflora* soil can absorb more heat during seasonal changes, while *Phragmites australis* soil is more affected by the terrestrial climate [23]. The temperature of *Phragmites australis* soil was lower in summer but higher in winter, because the soil surface was covered with snow or dead leaves, and heat preservation was stronger.

In the three seasons studied, the SH values at the HB site (36.7%) were all lower than those at the ZY and KA sites, and the values were highest (42.4%) at the KA site. The higher SH at the KA site may be due to the developed root system of *Phragmites australis* soil, as well as its strong water retention ability. Chambers et al. reported that although the *Phragmites australis* soil was at a higher tidal elevation and the flood duration was shorter, the soil in the *Phragmites australis* area can still maintain higher humidity [4]. However, *Spartina alterniflora* has shallow roots, a limited water holding capacity, and relatively low soil humidity.

The ranking of soil EC at the three sites was as follows: winter > autumn > summer. The impact of precipitation on soil salinity is seasonally regulated. It is difficult to leach the salt in the soil due to the decreased precipitation in winter, resulting in salt accumulation and higher EC. However, there is more precipitation in summer, and the salt is leached with precipitation. Therefore, the EC is reduced. Cebas-Csic et al. found that the seasonal dynamics of soil conductivity are closely related to precipitation in salt marshes in the semi-arid climate of southeast Spain [24]. In the arid period, soil salt accumulated due to the lack of leaching, and the conductivity increased. In the rainy season, the salt decreased, and the conductivity decreased due to precipitation leaching, which is consistent with the seasonal variation in conductivity observed in this study. In winter, the soil EC reached the highest value of 368.8 µs/cm at the KA site, and the lowest value of 243.2 µs/cm was measured at the HB site. The *Phragmites australis* at the KA site has developed roots and a strong water holding capacity. Sufficient water can fully dissolve the salt retained in the soil, resulting in high EC. The HB site is located in the offshore *Spartina alterniflora* area, and the shallow root system leads to a weak water holding capacity. Although the overall soil salinity is high, the ion concentration in the soil solution does not increase significantly, which may be due to the limited ion migration, resulting in the lowest EC. Zhang et al. observed that *Spartina alterniflora* had the characteristics of salt excretion in the wetland of the Yellow River Delta, and its invasion will reduce soil salt accumulation through its own physiological mechanism, thereby affecting soil EC [25].

### 3.2. Changes in NO_3_^−^, NO_2_^−^, NH_4_^+^, TOC, and Salinity in Different Soils

The contents of salinity, TOC, NO_3_^−^, NO_2_^−^, and NH_4_^+^ in the soils at the different sites are shown in Table 1. The salinity in the soil sampled from the HB site stood at 14.1 ± 1.04 g/kg, significantly higher than that at the ZY and KA sites. The differences in salinity may be influenced by the tides. The tide is frequent at the HB site, and the salt accumulates continuously. The ZY site is far from the sea; therefore, less seawater is present, and salt input decreases. Seawater has the lowest impact on the KA site. The KA site, characterized by precipitation and runoff dilution, has the lowest salinity value. The change in TOC was opposite to the salinity trend. The TOC content was highest (12.2 ± 0.76 g/kg) at the KA site and lowest (2.9 ± 0.39 g/kg) at the HB site. The root system of *Phragmites australis* penetrates deep into the soil at the KA site, and TOC components such as organic acids and phenols may be continuously input into the soil through root exudates [17]. However, the root system of *Spartina alterniflora* is shallow at the HB and ZY sites and influenced by tidal erosion. Some root exudates and leaves are easily taken away by seawater. Therefore, TOC accumulation efficiency is significantly lower than that of *Phragmites australis*. Zhang et al. proved that the promotional effect of *Spartina alterniflora* on soil TOC retention was insufficient due to the input of root resources, and the TOC accumulation efficiency was significantly lower than that of *Phragmites australis* [17]. The pH values of the three sites were alkaline, ranging from 7.6 to 8.0.

The concentrations of NO_3_**^−^** had no significant difference at the three sites. The concentration of NO_2_**^−^** ranged from 0.2 to 0.5 mg/kg, and the highest value, 0.5 mg/kg, was found at the ZY site, being higher than that at the HB and KA sites. Weingarten et al. found that salinity can affect nitrogen cycle processes induced by microorganisms, such as nitrification and denitrification [26]. A high-salinity environment may inhibit the activity of nitrifying microorganisms. The types of vegetation further affect nitrogen conversion efficiency by regulating soil nutrient supply and microbial community structure. The ZY site is different from the high-salinity environment of the HB site, and it does not have sufficient TOC and NH_4_^+^ like the KA site, which may lead to higher NO_2_**^−^** content. The concentration of NH_4_^+^ at the KA site was higher than that at the HB and ZY sites. *Phragmites australis* is a perennial herb with developed roots. Its preference for NH_4_^+^ utilization is lower than that of *Spartina alterniflora* at the HB and ZY sites, leading to a decrease in the consumption of NH_4_^+^ in soil. Windham-Myers compared the distribution of NH_4_^+^ in *Phragmites australis* and *Spartina alterniflora*, sampled from a brackish freshwater marsh, and found that *Spartina alterniflora*’s soil NH_4_^+^ absorption efficiency was higher than that of *Phragmites australis* [16].

### 3.3. Differences in Microbial Community Composition

**Figure 3:**
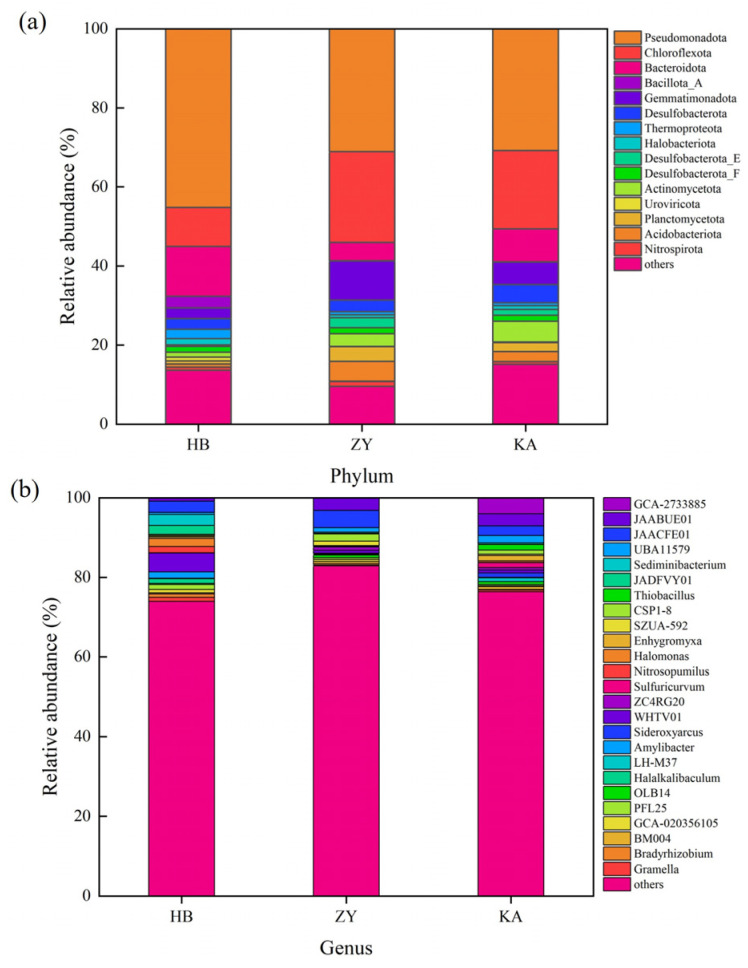
The relative abundance of microbial composition in three soil sites at phylum level (**a**), and at the genus level (**b**).

Metagenomic sequencing was performed to better understand the microbial composition and diversity in the different soils. The composition of soil microbial communities is shown in Figure 3. Differences in the composition of microbial genera between the three sites were apparent. At the phylum level, *Pseudomonadota*, *Bacteroidota*, *Chloroflexota*, *Gemmatimonadota*, and *Bacillota_A* were the dominant groups at the HB site, and the percentages of relative abundance were 45%, 13%, 10%, 3%, and 3%, respectively (Figure 3a). At the ZY site, *Pseudomonadota* (31%), *Chloroflexota* (23%), *Gemmatimonadota* (10%), *Bacteroidota* (5%), and *Acidobacteriota* (5%) were the major bacterial phyla. At the KA site, *Pseudomonadota* (31%), *Chloroflexota* (20%), *Bacteroidota* (8%), *Gemmatimonadota* (6%), and *Actinomycetota* (5%) were the major bacterial phyla. Gao et al. reported that *Pseudomonadota* was one of the most abundant phyla in *Spartina alterniflora* soil, and the relative abundance of *Bacteroidota* was also significantly higher than that of bare mudflat soil before invasion [5]. The relative abundance of *Pseudomonadota* was 31% in the ZY and KA sites, with this being slightly lower than that at the HB site, while *Bacteroidota* was less abundant at the ZY and KA sites. Furthermore, at the ZY and KA sites, *Chloroflexota* and *Gemmatimonadota* were more abundant than that at the HB sites. Studies have compared the effects of *Spartina alterniflora* and *Phragmites australis* on soil microorganisms and found that the input of living roots is the key factor driving the differences in microbial communities [17]. The root exudates of *Phragmites australis* contain higher concentrations of phenols and organic acids, which may selectively promote the growth of *Gemmatimonadota*.

**Figure 4:**
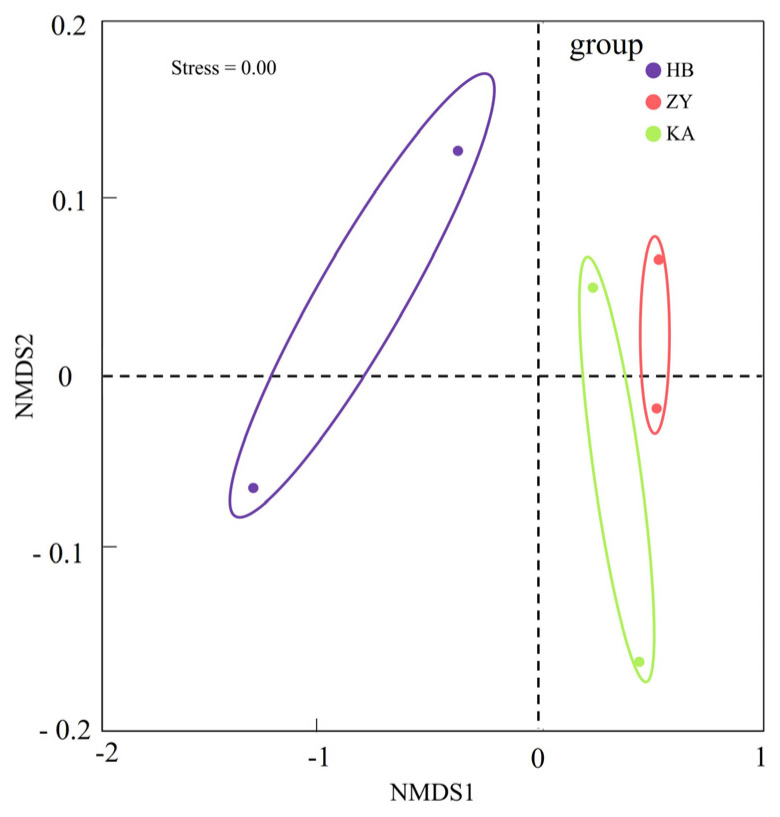
Nonmetric multidimensional scaling (NMDS) plot of the beta similarities measured as the Bray–Curtis distances at the three sites (*p* < 0.05).

At the genus level, *WHTV01*, *Sediminibacterium*, and *JAACFE01* were dominant at the HB site, and their relative abundance percentages were 5%, 3% and 3%, respectively (Figure 3b). At the ZY site, *JAACFE01*, *JAABUE01*, and *CSP1-8* were predominant, and their relative abundance percentages were 4%, 3%, and 2%, respectively. At the KA site, *GCA-2733885* (4%), *JAABUE01* (3%), and *JAACFE01* (2%) were dominant. Shan et al. found that the *Spartina alterniflora* soil was frequently affected by tides in the Yellow River Delta, forming a unique high-salt-high-organic matter microenvironment, which may provide a habitat for microorganisms in the marine estuarine transition zone [15]. The spatial distribution of the microbial community composition was performed using NMDS analysis (Figure 4). The stress value provides a reliable measure for evaluating the results of the NMDS analysis. If the stress value is less than 0.1, it can be considered well represented. NMDS analysis further proved that there were significant differences in microbial community composition among the three sites (*p* < 0.05).

### 3.4. Alpha Diversity of Soil Microbial Communities in Different Soils

**Figure 5:**
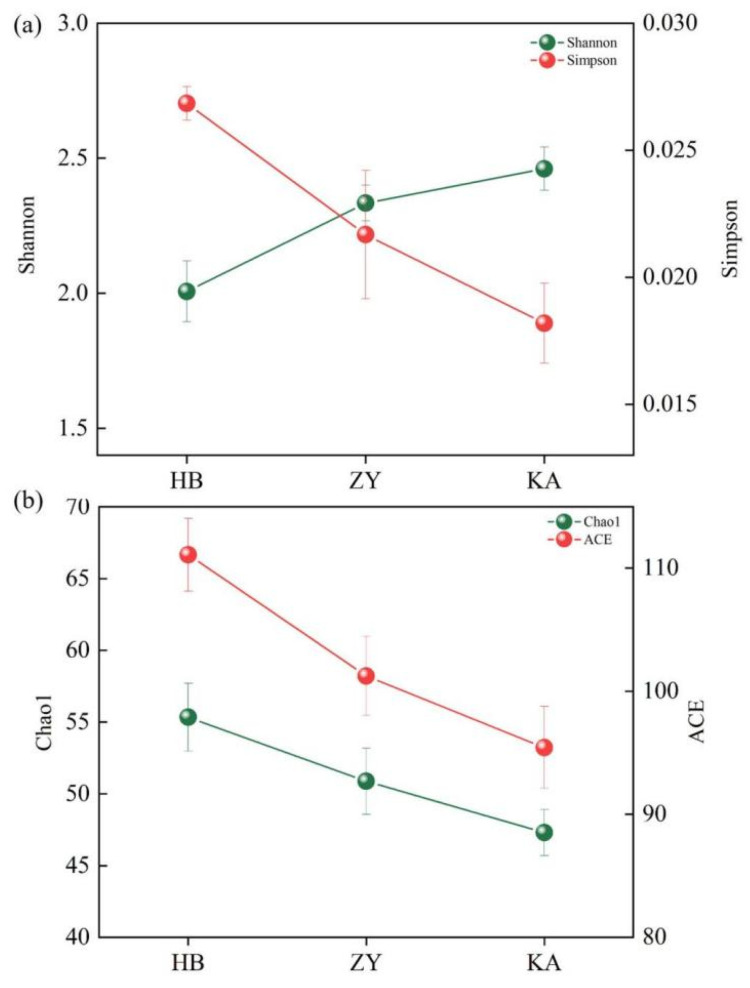
The microbial alpha diversity at three sites. Shannon index and Simpson index (**a**); Chao 1 index and ACE index (**b**).

The alpha diversity of soil microbial communities at different sites is shown in Figure 5. The Shannon index was the lowest (2.0) at the HB site and highest (2.4) at the KA site, indicating that the microbial community diversity of soil was the most uniform at the KA site. The Simpson index showed the opposite trend, with the highest value (0.26) being measured at the HB site and the lowest value (0.18) at the KA site. The changes in the Chao1 index and ACE index were similar to the Simpson index results, indicating species richness. The HB site is affected by tides, having the highest salinity value (Table 1). Frequent tides may bring a large number of marine microorganisms. Korbel et al. also found that there were frequent material and energy exchanges between living and non-living things in seawater, which resulted in an increase in the Chao1 and ACE indexes related to species richness [27]. A high-salt environment will inhibit the growth of most microorganisms, and only a few salt-tolerant groups are dominant [28], leading to poor community uniformity. Therefore, the Shannon index was the lowest, and the Simpson index was the highest at the HB site. However, at the KA site, the Shannon index was the highest, while the Chao1 and ACE indexes were the lowest. The KA site is a *Phragmites australis* habitat, characterized by a low salinity, high TOC, and high NH_4_^+^. It may provide rich carbon and nitrogen sources for microorganisms, which may lead to good uniformity in the microbial community. Moreover, the KA site is far away from the sea, meaning there are fewer external microorganisms brought by tides. Species competition in stable habitats tends to be balanced, potentially explaining the low values of species richness.

### 3.5. Correlation Between Soil Environmental Factors and Microbial Diversity

**Figure 6:**
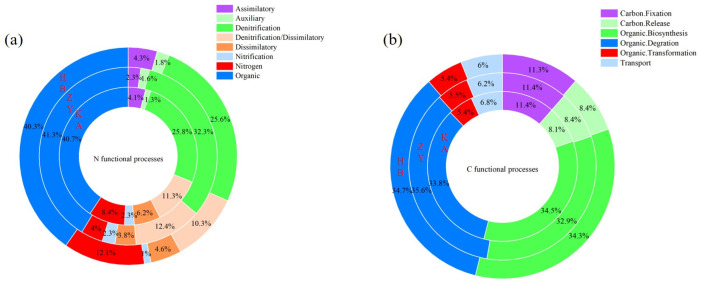
The relative abundance of N-cycle genes (**a**) and C-cycle genes (**b**) in microbial-mediated C-N cycle profiles in coastal wetlands.

The distribution of microbial carbon and nitrogen cycle function is shown in Figure 6. Nitrogen fixation was higher (12.1%) at the HB site than that at the ZY (4%) and KA (8.4%) sites (Figure 6a). The high-salt environment of the HB site may screen out salt-tolerant nitrogen-fixing microorganisms, and the low NH_4_^+^ at this site leads to soil nitrogen deficiency. Microorganisms need to supplement nitrogen sources through nitrogen fixation, so the nitrogen fixation process is clearest at the HB site. The results were consistent with the dominant bacterial community structure of *Pseudomonadota* and *Bacteroidota* at this site (Figure 3a). *Pseudomonadota* and *Bacteroidota* have been reported to possess nitrogen fixation and organic nitrogen decomposition functions. There are diverse salt-tolerant nitrogen-fixing microbes in these two phyla, capable of adapting to high-salinity coastal habitats [2,9]. The proportion of denitrification/dissimilatory nitrate reduction was 11.3% at the KA site, 12.4% at the ZY site, and 10.3% at the HB site, respectively. There was no significant difference across the three sites. The proportion of the dissimilatory nitrate reduction process was higher at the KA site (6.2%), compared to the HB (4.6%) and ZY (3.8%) sites. The KA site, with its low salt content, may relieve the inhibition of salt ions on dissimilatory nitrate-reducing microorganisms and provide sufficient energy due to the high-TOC environment. The root exudates of *Phragmites australis* may also selectively promote the microbial community of dissimilatory nitrate-reducing bacteria, especially *Chloroflexota* and *Gemmatimonadota*, which were relatively enriched in the KA site (Figure 3a). *Chloroflexota* and *Gemmatimonadota* are associated with the processes of nitrification, denitrification, and dissimilatory nitrate reduction in coastal wetland ecosystems [5,12]. Therefore, these processes have the highest proportions. The proportions of organic degradation and organic biosynthesis were, respectively, 33.8% and 34.5% at the HB site, 35.6% and 32.9% at the ZY site, and 34.7% and 34.3% at the KA site (Figure 6b). *Pseudomonadota*, *Bacteroidota*, *Chloroflexota*, and *Gemmatimonadota* have been proven to participate in the decomposition and synthesis processes of organic matter [29,30,31]. The differences in carbon release and carbon fixation processes among the three sites were negligible, standing at 8.1–8.4% and 11.3–11.4%, respectively.

**Figure 7:**
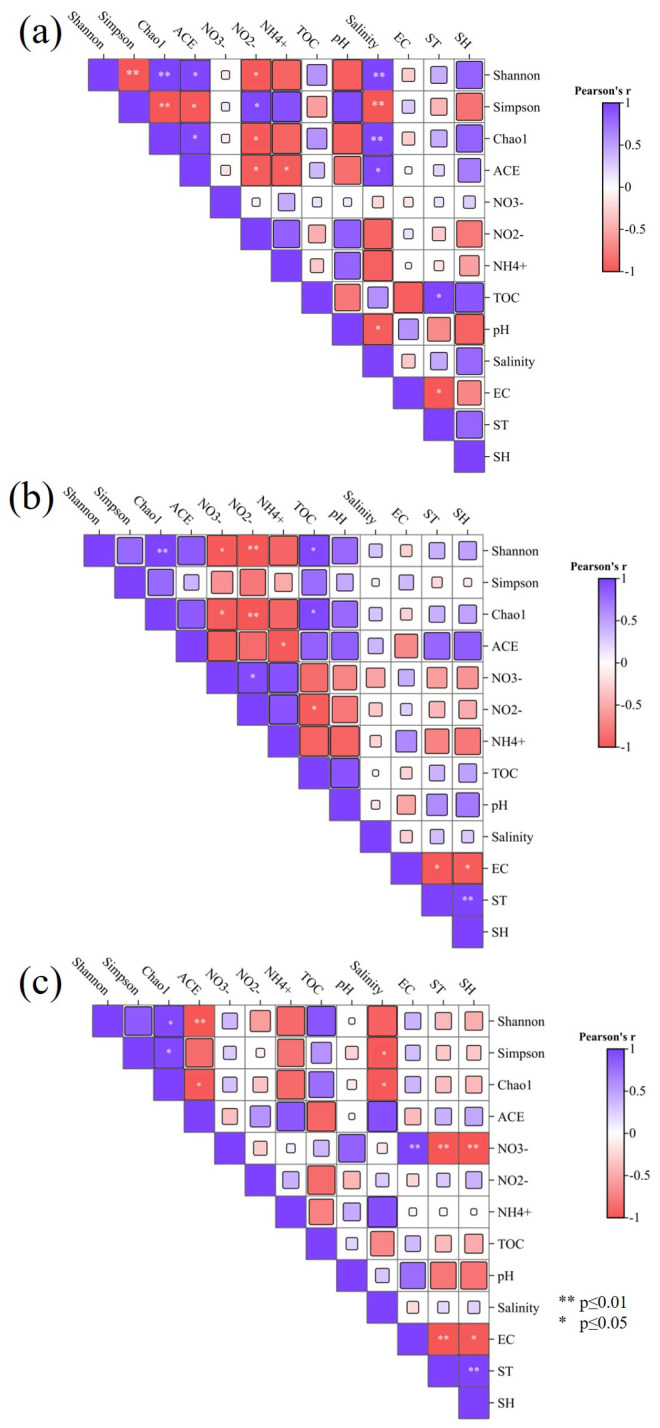
The heat map of correlation between microbial diversity indexes and environmental factors at HB site (**a**), ZY site (**b**), and KA site (**c**) of coastal wetlands.

The correlation between soil environmental factors and the microbial diversity index is shown in Figure 7. Salinity was correlated with the Shannon and Chao1 indexes (r = −0.99, *p* < 0.05), Simpson index (r = 0.99, *p* < 0.05), and ACE indexe (r = −0.96, *p* < 0.05) at the HB site. NO_2_^−^ was also significantly correlated with the Shannon, Simpson, Chao1, and ACE indexes (*p* < 0.05). The results indicate that salinity has a strong impact on the uniformity and species richness of the microbial community. At the ZY site, TOC and NO_3_^−^ were correlated with the Shannon and Chao1 indexes (r = 0.96, *p* < 0.05). NO_2_^−^ was also correlated with the Shannon and Chao1 indexes (*p* < 0.05). The Chao1 index and the Shannon index showed a significant positive correlation (r = 0.99, *p* < 0.01). The ZY site is situated between the HB and KA sites; therefore, the environmental factors, such as salinity and TOC, were more moderate at the ZY site. When the Chao1 index increased, the new species may co-exist with the original groups stably, and the dominant groups would not monopolize resources, resulting in a simultaneous increase in the Shannon index of community uniformity. At the KA site, salinity was correlated with the Simpson index (r = −0.93, *p* < 0.05) and the Chao1 index (r = −0.98, *p* < 0.05). Meanwhile, ST (r = 0.99, *p* < 0.05) and EC (r = −0.98, *p* < 0.05) were correlated with SH, and the Shannon index was significantly negatively correlated with the ACE index (r = −0.99, *p* < 0.01). At the KA site, *Phragmites australis* has a strong water holding capacity and good heat preservation properties. This site had stable, high soil humidity throughout the year; therefore, temperature and humidity changes occurred synchronously. At the same time, temperature and humidity had a strong impact on EC at this site. Therefore, salinity, total TOC and soil humidity may be the key environmental factors that influence the microbial diversity in the study area.

## 4. Conclusions

We studied the microbial community structure and major environmental factors in different soils sampled from a specific coastal wetland. The HB site is frequently affected by tides, and it is the site with the highest salinity. The tidal input of marine microorganisms leads to the highest Chao1 and ACE indexes related to species richness. However, the high-salt environment may inhibit the growth of most microorganisms, and a few salt-tolerant groups may be dominant, which may reduce the uniformity of the microbial community. *Spartina alterniflora* may cause low soil humidity and low EC at the HB site. Low salinity and higher TOC and NH_4_^+^ contents were identified in the soils at the KA site. *Phragmites australis* has developed roots, as well as a strong water retention capacity. Low-salt conditions relieve the inhibition of salt on microorganisms, and sufficient carbon and nitrogen sources provide a good growth environment for microorganisms. Therefore, the microbial community uniformity (Shannon index) was high. As a transitional habitat of *Spartina alterniflora*, the ZY site has moderate environmental pressure. The indexes of microbial diversity were at the middle level. Furthermore, there were significant differences in microbial community composition among the three sites. *Pseudomonadota*, *Bacteroidota*, *Chloroflexota*, and *Gemmatimonadota* are the predominant microbial components. Salinity, total TOC and soil humidity may be the key environmental factors that influence the microbial community in the study area. They can change the soil microenvironment to screen specific microbial groups and affect carbon and nitrogen cycling processes, including nitrogen fixation, dissimilatory nitrate reduction, and organic degradation.

## Figures and Tables

**Table 1:** Physicochemical properties of different soils in coastal wetlands.

Sites	NO_2_^−^ (mg/kg)	NO_3_^−^ (mg/kg)	NH_4_^+^ (mg/kg)	TOC (g/kg)	pH	Salinity (g/kg)
HB	0.3 ± 0.07	11.8 ± 1.3	14.0 ± 0.8	2.9 ± 0.39	7.9 ± 0.08	14.1 ± 1.04
ZY	0.5 ± 0.04	12.0 ± 1.7	20.0 ± 2.9	4.9 ± 1.19	8.0 ± 0.02	5.9 ± 0.91
KA	0.2 ± 0.01	11.0 ± 1.9	37.0 ± 1.9	12.2 ± 0.76	7.6 ± 0.01	4.4 ± 0.63

## Data Availability

The original contributions presented in this study are included in the article. Further inquiries can be directed to the corresponding authors.
